# Memorial of Herr Theodore Mehlhardt

**Published:** 1880-06

**Authors:** W. Sachs, C. H. E. Obermuller

**Affiliations:** Breslau.


AMERICAN JOURNAL

OF

### DENTAL SCIENCE.

Vol. XIV. THIRD SERIES—JUNE, 1880. No. 2.

### ARTICLE I.

[The memorial of Mr. Mehlhardt was presented to the German Emperor and Parliameut on the occasion of the Golden Wedding of the Emperor.]

### *A Few Remarks about the Memorial of Herr Theodore Mehlhardt.*

BY DR. W. SACHS, BRESLAU.

[Translated from the *Correspondenz Blatt*, January No., 1880, Page 50. BY DR. C. H. E. OBERMULLER.]

Mr. Mehlhardt talks in this manner about American dentistry in a very low degree so that those people that are not acquainted with the circumstances, between the real American dentist and Zahntechnicker (persons in Germany who practice but are not recognized by the Government, or by any diploma—translator) are deceived. The standpoint of the author will not be so readily recognized from all good standing dentists and will prove that the old gentleman stands very far behind in dental science in the last thirty years.

Dr. Sachs gives something from Mehlhardt, Page 10. M. says: "We will not hesitate to say that the dental mechanism to Americans has made very important progress.

Through the filling of carious teeth, many people will keep the teeth longer, and maybe useful for mastication. But sorry to say very greater number are uselessly maltreated under great pain and trouble, cheated out of their money, and afterwards the filled teeth have to be, in many cases extracted any way."

About these words will every dentist smile because Mr. Mehlhardt believes the filling of teeth does more damage than good, and Mehlhardt says also that it is American dentists who brought about the filling of teeth into general use. Now on Page 11, we read further: "The Dental instruments are too often in the wrong hands, especially in the hands of dentists who have graduated in America are instruments of martyrdom. And the consequence of this is that all the teeth of the people are ruined."

It is certain that dental instruments in the wrong hands may do immense damage, but why is it just American dentists in whose hand is this damage. I believe that incompetence is an international property, and not confined to American dentists only. Pages 11 to 14, Mehlhardt gives some cases from his practice: "Twelve or thirteen years ago a young lady aged 22 to 25 years came to me who had previously all her teeth and roots extracted by a distinguished American dentist, for the purpose of having artificial teeth inserted. I declined to make her an artificial set because it is against my custom to extract sound teeth and roots, (tight setting.) I told her I was very sorry for the lady but I declined to make her an artificial set and sent her back to the distinguished American dentist who extracted the teeth. Although I know this gentleman has no knowledge about dental surgery, and I could not as man and specialist, send any one to him for treatment; but I told the lady in case she had any trouble I was ready to give her medical treatment, but I would not make the teeth."

Now I (Dr. Sachs,) ask from what knows Mr. Mehlhardt that the extraction of the roots and teeth was not necessary for the condition of the mouth, because when the patients came to him some months were past since the extraction of the teeth. And even in the case the American dentist extracted teeth which ought not to have been extracted, the blame against American dentistry can not be, because in Germany such an American dentist lives and does such malpractice. When Mr. Mehlhardt believes that the wholesale extraction of teeth is taught in American Colleges, he is very much mistaken. It is the principle of every good honest American dentist to preserve to the patient every tooth and root that has any usefulness.

Second case on page 12, Mr. Mehlhardt refers to, seems to be a very hard one, because it is not only a lower set, which is also made by a *distinguished American Dentist*, and did not fit, but particularly Mr. Mehlhardt says that a whole family are ruined by this artificial piece.

Further Mr. Mehlhardt says that American Colleges grant diplomas to young men at 18 years of age. As far as I know American Colleges grant diplomas to those only who have passed their 21st year, and hence the diplomas of this young man must be false. And from the foregoing it is evident that the author has very wrong provocation against American Dentists. When one or more American Dentists are known to him as incompetent and as such men who do not look at a patient to give relief, but only to make as much money out of them as possible, so should he not make that a fault of American Surgery. If Mr. Mehlhardt would take the trouble to make himself acquainted with the practice of other American dentists, he would soon find out that he could learn a good deal from them, and as long as we have not a German Dental College controlled by the State after the suggestions of Dr. G. Wm. Lamdorff, (*Viertellyapropyoft*, 1877, page 436.) I would advise every young man who wanted to study dentistry to go to America after he has finished his studies in the German University. There he finds what we need here—Dental Colleges, who give all those men who want to learn all that is necessary to become competent dentists who stand the height of their profession. I do not intend to say that nobody can become here a good dentist, but he must seek a chance in his education, while in America he can seek it in full. So much for American Dentists.

Mr. Mehlhardt discovered that better teeth the people have, the better they can pay the taxes. Mr. Mehlhardt says on page 8: "To-day there are a good deal of remedies to give relief from tooth-ache, all with success. But as experience teaches, it has been, and will be nothing but trying by which the pain mostly increases and at least there is no other way when the inflammation has come to its highest state, by neglect or false treatment to relieve but by extracting the tooth."

Again on page 15, Mr. Mehlhardt says: "It has always been a mistake to extract the peoples' teeth, on account of tooth-ache, because every tooth can be cured without extraction of the tooth, especially when the patient right away after feeling the pain, seeks relief from a competent educated dentist." I ask here: How will Mr. Mehlhardt explain this.

On page 8, Mr. Mehlhardt says: "At the commencement of this century, the carious teeth were a great deal more numerous. Of 100 people at the age of 40; 50 per cent. had a useful set of teeth. Now only 10 per cent. of that number have a useful set. Already at the age of 30, 9-10ths of the present generation are teeth patients, and of this we can judge from good authority that the next generation will wear in their 30th year, mostly artificial teeth. In other words, *are cripples as regard digestion*, if dentists keep up their present system."

So rapid have carious teeth not progressed, as Mr. Mehlhardt says. On the contrary I say that in the last 25 years, less teeth are lost than in previous years. The public is kept informed by good dentists about the mouth and how to preserve the teeth, so the next generation will not be "cripples in digestion" if the present system is carried on, but will have better teeth. Because from their youth they will be taught to preserve them and by themselves, contribute to their preservation.

Mr. Mehlhardt believes that the filling of temporary teeth is not only useless but absolutely dangerous. So they taught 30 years ago, but to-day every competent dentist seeks to preserve the teeth of the little ones, in order to spare them pain, and in order to preserve the temporary teeth until the permanent ones are ready to come through.

Mr. Mehlhardt would do the suffering humanity invaluable service if he would tell us the method by which he fills carious teeth exactly painless to the patient; and in which way he treats every toothache without extracting the tooth.

There is a good deal said in this important paper (?) of Mr. Mehlhardt, which has been written by a gentleman who has enjoyed a very large practice of 30 years. But I believe that hardly all that is said in this paper will be of any benefit to suffering humanity.

---

